# Predict Colon Cancer by Pairing Plasma miRNAs: Establishment of a Normalizer-Free, Cross-Platform Model

**DOI:** 10.3389/fonc.2021.561763

**Published:** 2021-04-22

**Authors:** Da Qin, Qingdong Guo, Rui Wei, Si Liu, Shengtao Zhu, Shutian Zhang, Li Min

**Affiliations:** Department of Gastroenterology, Beijing Friendship Hospital, Capital Medical University, National Clinical Research Center for Digestive Disease, Beijing Digestive Disease Center, Beijing Key Laboratory for Precancerous Lesion of Digestive Disease, Beijing, China

**Keywords:** colon adenocarcinoma, miRNA, circulation miRNA, miRNA-pair, miRNA standardization

## Abstract

**Background:**

Plasma miRNAs are emerging biomarkers for colon cancer (CC) diagnosis. However, the lack of robust internal references largely limits their clinical application. Here we propose a ratio-based, normalizer-free algorithm to quantitate plasma miRNA for CC diagnosis.

**Methods:**

A miRNA-pair matrix was established by pairing differentially expressed miRNAs in the training group from GSE106817. LASSO regression was performed to select variables. To maximize the performance, four algorithms (LASSO regression, random forest, logistic regression, and SVM) were tested for each biomarker combination. Data from GSE106817 and GSE112264 were used for internal and external verification. RT-qPCR data acquired from another cohort were also used for external validation.

**Results:**

After validation through four algorithms, we obtained a 4-miRNA pair model (miR-1246 miR-451a; miR-1246 miR-4514; miR-654-5p miR-575; miR-4299 miR-575) that showed good performance in differentiating CC from normal controls with a maximum AUC of 1.00 in internal verification and 0.93 in external verification. Tissue validation showed a maximum AUC of 0.81. Further external validation using RT-qPCR data exhibited good classifier ability with an AUC of 0.88.

**Conclusion:**

We established a cross-platform prediction model robust against sample-specific disturbance, which is not only well-performed in predicting CC but also promising in the diagnosis of other diseases.

## Introduction

With 1.9 million new cases, colon cancer (CC) ranks third among all cancers in incidence worldwide (1). The number of CC patients grows rapidly during the past decade, which has become a major global health problem (2). Early-stage CC patients could be cured by minimally invasive surgery and showed a 5-year survival rate of 74% (3). Thus, detection of CC at a resectable stage is a long-term pursuit of gastroenterologists (4).

Plasma microRNAs (miRNAs), representing an emerging direction of liquid biopsy, are bringing new insights to early detection of CC. Many efforts have been made to evaluate plasma miRNAs to predict CC (5, 6). Among the main existing miRNA detection methods, omics tools such as miRNA-sequencing and miRNA chip are costly and not suitable for large-scale population cancer screening. Real-time quantitative PCR (RT-qPCR) is a cost-effective alternative to the omics tools in miRNA detection. However, the absence of a proper standardization method for RT-qPCR data of plasma miRNA largely limited its reliability and repeatability in quantification assays (7). Thus, although dozens of miRNA biomarkers have been proposed in different labs, few of them were reproducible by other researchers and could eventually be applied to large-scale population screening of cancers.

In this study, we proposed a miRNA paired ratio-based standardization method for plasma miRNA quantification. We calculated the ratio of two given miRNAs in the same plasma sample, used this ratio as a new kind of variables, and constructed a prediction system based on these ratios to predict risks of CC. This ratio-based system performed well in both miRNA chip data and RT-qPCR data by eliminating possible sample-specific disturbance. We believe that this system is not only well-performed in predicting CC but also promising in the diagnosis of other diseases.

## Materials and Methods

### Patients Cohorts

**Figure 1:**
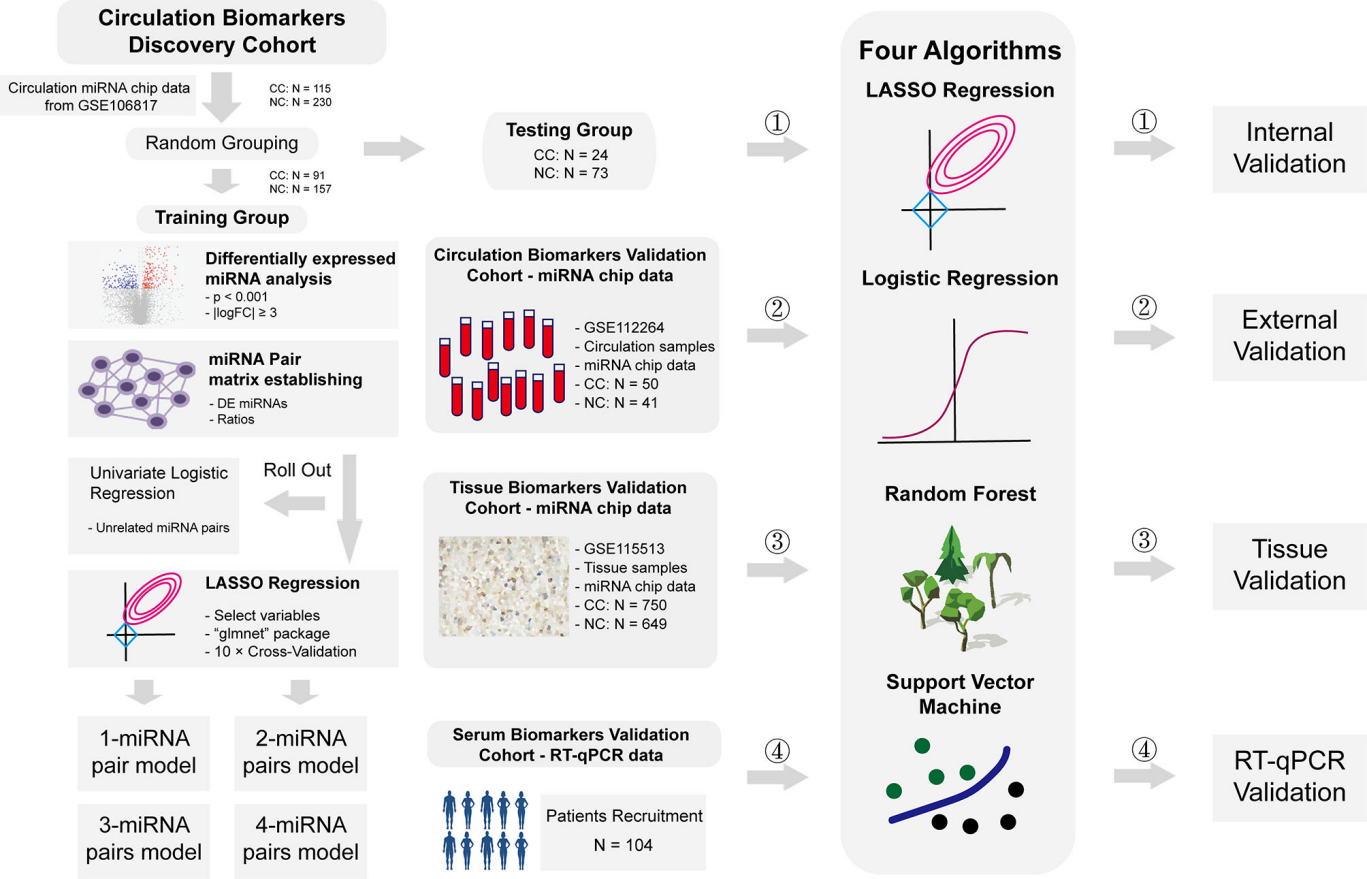
Flowchart of study. Flowchart of this study. In short, we calculated the ratio of two given miRNAs in the same plasma sample, used this ratio as a new kind of variables, constructed a prediction system based on these ratios to predict risks of CC, and validated these models in internal level, external level, tissue level and RT-qPCR level.

Plasma miRNA chip data from GSE106817 (8) and GSE112264 (9) in GEO database were obtained for analysis. GSE106817 was divided into a training group and a testing group by random number. GSE112264 was used for external verification. Furthermore, miRNA chip data of tissue samples from GSE115513 (10) was also used for validation in tissue samples. 104 plasma samples of CC patients and normal controls from Beijing Friendship Hospital were collected for RT-qPCR data validation. Clinical details of these subjects were shown in **Supplementary File 1**. All participants had signed the informed consent, and this study was approved by the ethics committee of Beijing Friendship Hospital. Flowchart of this study was shown in **Figure 1**.

### Differentially Expressed miRNA Analysis and miRNA-Pair Matrix Construction

Differentially expressed (DE) miRNA analysis was performed to training group derived from GSE106817 using “limma” package (11) in R 3.5.2. Then, miRNA paired ratios were calculated from any two DE miRNAs expression values in the same sample. Subsequently, univariate logistic regression was used to exclude pairs not associated with cancer occurrence and constructed a final miRNA-pair matrix for the training group.

### Prediction Model Construction

LASSO regression was performed to select variables in the miRNA-pair matrix using “glmnet” package (12) in R 3.5.2. Models enrolled 1, 2, 3, 4 miRNA-pairs were successively established by a step-wise variable selection process by controlling lambda values in a LASSO regression. Then, the logistic prediction model for each of these models were constructed by “glm” function in R. Random forest models were established by “randomForest” package. Support vector machine (SVM) models were designed using “e1071” package. Furthermore, area under the curve (AUC) of all these prediction models were calculated to evaluate their performance.

### Internal and External Verification by miRNA Chip Data

The testing group of GSE106817 was used for internal verification of the models. Receiver operator characteristic (ROC) curves of these models were drawn by using “plotROC” package (13) and “ggplot2” package. GSE112264 was used for external verification of these models. Furthermore, we also evaluated the diagnostic validity of those models in tissue miRNA chip data from GSE115513. AUC values of internal and external verification for each model were calculated.

### RT-qPCR Validation

104 plasma samples from Beijing Friendship Hospital were used for external validation (**Supplementary File 1**). Total RNA isolated from plasma was obtained using TRIzol following the manufacturer’s protocol (Takara, Shiga, Japan). Total RNA was reverse-transcribed and cDNA was synthesized using miR-specific stem-loop RT primers and components of the High-Capacity cDNA Reverse Transcription kit (Takara, Shiga, Japan, RR036a). The amplification of cDNA was performed in 10-μl reaction system following the SYBRGREEN life assays manufacturer’s instructions. Primers used in RT-qPCR validation were shown in **Supplementary File 2**.

### GSEA Analysis, Gene Ontology Analysis and KEGG Pathway Analysis

Target genes of selected miRNAs were predicted by TargetScan (14). Gene ontology analysis was then performed to identify ontologies (MF, molecular functions; BP, biological processes; CC, cellular component) enriched in the target genes of those miRNAs using clusterProfiler (15) package in R software. KEGG pathway and GSEA enrichment analysis (16) was also performed to find out the potential pathways which may be affected by those miRNAs.

### Statistical Analysis

All statistical analyses were calculated by R software (version 3.5.2; https://www.r-project.org/). DE miRNAs were identified by “limma” package (version 3.38.3). ROC curves were graphed using “plotROC” package (version 2.2.1). LASSO regression was performed using “glmnet” package (version 2.0-18). Random forest regression and SVM were performed using “randomForest” package (version 4.6-14) and e1071 package (version 1.7-2). Statistical significance was defined as P <0.05.

## Results

### DE miRNA Identification and miRNA-Pair Matrix Construction

387 DE miRNAs were identified after DE analysis in the training group (91 CC and 157 NC) of GSE106817 (**Figure 1**). Any two of these 387 DE miRNAs were paired, which constructed a miRNA-pair matrix containing 74,691 miRNA-pairs. Univariate logistic regression was subsequently performed to screen for cancer-related miRNA-pairs. Finally, a miRNA-pair matrix with 61,939 miRNA-pairs was obtained.

### Prediction Model Construction and Internal Verification by miRNA Chip Data

**Figure 2:**
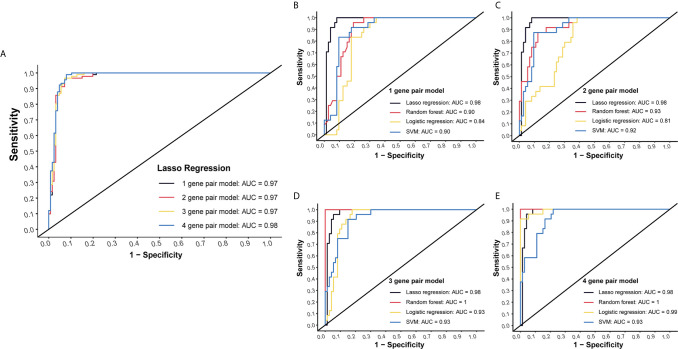
Internal validation of four models by four methods. **(A)** Models included 1, 2, 3, 4 miRNA-pairs were established successively by a step-wise variable selection process in a LASSO regression. AUC levels of them were all more than 0.97. **(B)** Internal validation of 1-gene pair model using LASSO regression, random forest, logistic regression and SVM methods. **(C)** Internal validation of 2-gene pair model. **(D)** Internal validation of 3-gene pair model. **(E)** Internal validation of 4-gene pair model.

LASSO regression was performed to select the most effective variables from all miRNA-pairs to construct multivariate prediction models. Models included 1, 2, 3, 4 miRNA-pairs were established successively by a step-wise variable selection process in a LASSO regression, which is shown in **Table 1** (**Figure 2A**).

**Table 1:** Gene models established by LASSO regression.

miRNA-pair models	miRNA-pairs	coefficient	Lambda	AUC
1-miRNA-pair model	hsa-miR-1246/hsa-miR-451a	0.031	0.32	0.9683
2-miRNA-pair model	hsa-miR-1246/hsa-miR-451a	0.170	0.23	0.9686
	hsa-miR-1246/hsa-miR-4514	0.004		
3-miRNA-pair model	hsa-miR-1246/hsa-miR-451a	0.206	0.2	0.9719
	hsa-miR-1246/hsa-miR-4514	0.034		
	hsa-miR-654-5p/hsa-miR-575	0.0003		
4-miRNA-pair model	hsa-miR-1246/hsa-miR-451a	0.223	0.18	0.9753
	hsa-miR-1246/hsa-miR-4514	0.059		
	hsa-miR-654-5p/hsa-miR-575	0.002		
	hsa-miR-4299/hsa-miR-575	6.075		

Plasma miRNA chip data of 24 CC and 73 NC from the testing group was used for internal validation. To fully maximize the performance of these models, four methods (LASSO regression, random forest, logistic regression and SVM) were simultaneously tested. High AUC results of at least 0.9 in each model were obtained, which suggested a good efficacy in CC detection (**Figures 2B–E**).

### External Verification of Prediction Models by miRNA Chip Data

**Figure 3:**
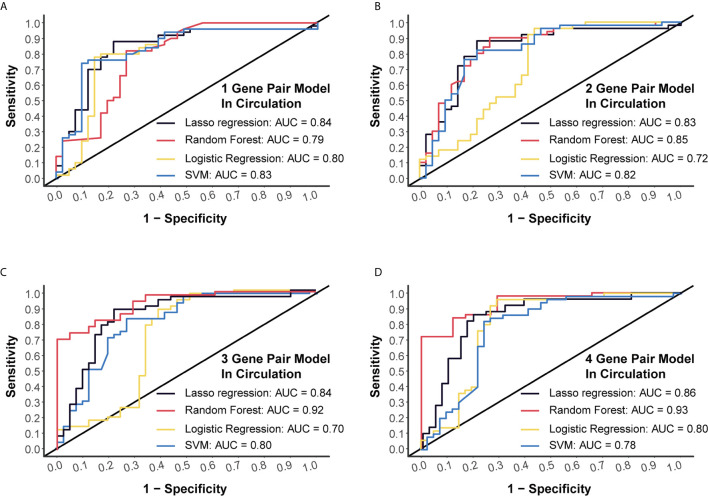
External validation of four models using miRNA chip data. **(A)** External validation of 1-gene pair model using LASSO regression, random forest, logistic regression and SVM methods. **(B)** External validation of 2-gene pair model. **(C)** External validation of 3-gene pair model. **(D)** External validation of 4-gene pair model.

Another dataset (GSE112264) with plasma miRNA chip data of 50 CC and 41 NC was used for external verification. Four algorithms (LASSO regression, random forest, logistic regression and SVM) were applied to validate each of these models, and we finally obtained high AUC levels of over 0.7 in each model (**Figures 3A–D**). Especially, for the 4-miRNA-pair models, AUC of different algorithms was 0.8556, 0.9346, 0.7985, 0.7759, respectively (**Figure 3D**). Those results suggested a good reproducibility of our models in different cohorts.

### Tissue Verification of Prediction Models by miRNA Chip Data

**Figure 4:**
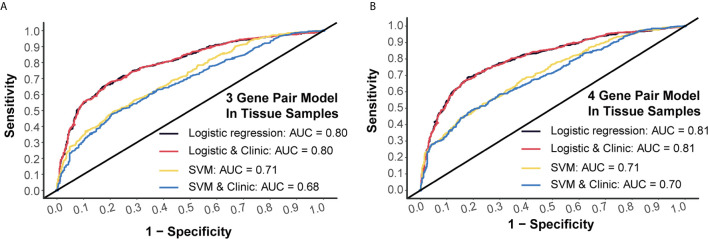
External validation of prediction models in tissue samples. **(A)** Tissue validation of 3-gene pair model using logistic regression and SVM methods. **(B)** Tissue validation of 4-gene pair model using logistic regression and SVM methods.

Generally, 3-miRNA-pair models and 4-miRNA-pair models with an AUC >0.8 could be considered as possibly applicable in clinical scenarios. To better understand their rationality, we further validated those constructed models in a tissue sample-derived miRNA chip dataset (GSE115513). For 3-miRNA-pair models, the SVM method could reach an AUC value of 0.71, while the AUC value of the logistic regression was 0.80 (**Figure 4A**). For 4-miRNA-pair models, the logistic regression method could reach an AUC value of 0.81 (**Figure 4B**). Those results exhibited reliable diagnostic performance of our prediction models in tissue samples.

### Validation for Potential Clinical Application by RT-qPCR

**Figure 5:**
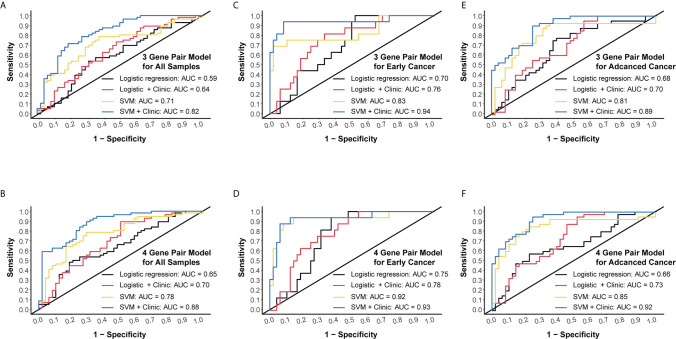
External validation and subgroup analysis of selected models using plasma miRNA q-PCR data from Beijing Friendship Hospital. **(A)** External validation of 3-gene pair model using logistic regression and SVM methods. Red and blue lines represent adding patients’ age and gender into consideration. **(B)** External validation of 4-gene pair model. **(C)** ROC assessment of 3-gene pair model predicting early cancer. **(D)** ROC assessment of 4-gene pair model predicting early cancer. **(E)** ROC assessment of 3-gene pair model predicting advanced CC. **(F)** ROC assessment of 4-gene pair model predicting advanced CC.

Next, we conducted RT-qPCR assays to validate those models. For the 3-miRNA-pair model, SVM method could reach an AUC value of 0.71, which could boost to 0.82 when taking clinical information such as age and gender into consideration. However, logistic regression models gave a lower AUC level as compared to SVM (**Figure 5A**). For the 4-miRNA-pair model, AUC of SVM was 0.78 and could increase to 0.88 when adding age and gender into this model (**Figure 5B**).

Then we performed subgroup analysis focusing on early CC patients and advanced CC patients, respectively. An AUC of 0.83 in the 3-miRNA-pair SVM model and an AUC of 0.92 in the 4-miRNA-pair SVM model were obtained when applying those models to distinguish 16 early CC patients from 48 normal controls (**Figures 5C, D**). Similarly, a sub-cohort consists of 40 advanced CC patients and 48 normal controls was also analyzed. The AUC reached to 0.81 in the 3-miRNA-pair SVM model and 0.85 in the 4-miRNA-pair SVM model (**Figures 5E, F**). The comparable efficiencies between detecting early CC and advanced CC are inspiring, but the sample size is too small to draw a definite conclusion.

### GSEA Analysis, GO Analysis and KEGG Pathway Analysis for the Involved miRNAs

**Figure 6:**
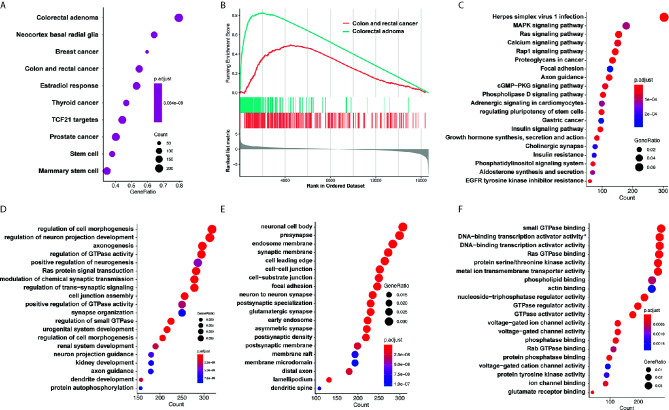
GO analysis and KEGG pathway analysis. **(A, B)** GSEA analysis showed target genes were mostly related to colorectal adenoma and colorectal cancer. **(C)** KEGG analysis showed that target genes of miR-4299 were mainly involved in HSV-1 infection and MAPK signaling pathway. DEF. GO analysis showed that these target genes were involved in regulation of cell morphogenesis **(D)**, neuronal cell body **(E)** and small GTPase binding **(F)**. *: RNA polymerase II−specific.

Target genes of selected miRNAs were predicted by TargetScan based on their seed positions, which were shown in **Table 2**. GSEA analysis was then applied and showed target genes were mostly related to colorectal adenoma and colorectal cancer (**Figures 6A, B**).

**Table 2:** Target gene analysis of miRNAs in prediction model.

miRNA	Target genes
hsa-miR-1246	CDR1as, FAM169B, GSG1L, ZNF23, ZNF267, ZNF83, MEIS3, ZFP69B, FAM53C, C12orf71
hsa-miR-451a	OSR1, ATF2, MIF, PSMB8, TSC1, S1PR2, C11orf30, AEBP2, GK, VAPA
hsa-miR-4514	AGO2, NBPF20, NBPF12, PRX, C19orf77, LCE1E, ZNF460, CYP4A22, NOTCH2, LCE1D
hsa-miR-654-5p	RSPO4, PRX, GUCA2B, GNG13, SYNDIG1L, PVRL1, FAM222B, PIP5K1C, PLEKHM1, RASD2
hsa-miR-575	ASPHD1, AC073610.5, HTN3, A4GNT, NANOGNB, AGAP2-AS1, BID, FOXRED1, PRSS46, REG4
hsa-miR-4299	ZNF256, ZIK1, SHISA7, MCTS1, ZNF584, BRIX1, SSMEM1, TEX12, ZNF772, DAZ4

KEGG analysis and GO analysis were also performed to find out gene functions and pathways affected by these miRNAs. KEGG analysis showed that target genes were mainly involved in HSV-1 infection and MAPK signaling pathway (**Figure 6C**). GO analysis showed that they were involved in regulation of cell morphogenesis (biological processes, **Figure 6D**), neuronal cell body (cellular component, **Figure 6E**), and small GTPase binding (molecular functions, **Figure 6F**).

## Discussion

Early detection of CC is clinically crucial but technically difficult, since the patients are hardly to exhibit any symptoms until turning into advanced stages (17). Traditional biomarkers (e.g., CEA and CA19-9) showed low sensitivity and specificity in cancer detection, which may cause missed and delayed diagnosis. Recently, an increasing number of researches focused on new biomarker types, such as plasma miRNAs (18). Plasma miRNAs are stable under various storage conditions and resistant to degradation, which made them good candidates for cancer biomarkers (6). For example, Vychytilova-Faltejskova et al. enrolled 144 colon cancer patients and 96 healthy controls, and sequencing 20 libraries each containing pooled RNA of 12 different cases (19). They constructed models by logistic regression and validated them in RT-qPCR data of 427 patients. This study had a large sample size and recorded many clinical details; however, only logistic regression was used for model construction, and the normalization method used in serum miRNA RT-qPCR quantification wasn’t provided. María et al. enrolled 123 patients (63 with CRC, 60 with advanced adenomas) and 73 healthy controls, and identified 6 plasma miRNAs for CRC detection, which also represented good classifier abilities to advanced adenomas (20). This study had large sample volume and selected a relatively stable miRNA (miR-16) as internal reference; however, a proper modeling algorithm could make its diagnostic value better.

Among all detection methods, RT-qPCR is a proper method for clinical application for its low-cost as compared to miRNA-seq and miRNA chips (21). However, its accuracy was often questioned due to the low concentration of miRNAs in plasma and no reliable normalizer for quantification. There are two regular methods for plasma miRNA standardization. The most popular method is using traditional reference genes, such as U6 (22) and miR-16 (23), which have been considered stably expressed in human cells. However, even those reference miRNA could be dysregulated and exhibited a altered abundance in the circulation system in a disease-specific manner (24, 25). Consequently, those reference RNAs are not suitable for data normalization in circulating miRNA quantification. Spike-in exogenous miRNAs such as cel-miR-39, cel-miR-54, SV40 were also used as external references to partially eliminate deviations from experimental processes. However, these external controls could not correct sample specific deviations (26). Here we proposed a miRNA-pair ratio-based algorithm to quantitate plasma miRNA. We calculated the ratio of any two candidate miRNAs in the same sample, and taking these ratios as new variables to predict cancer occurrence. Considering that expression levels of two miRNAs were simultaneously measured under same conditions, their ratio could reflect true fold change ratio by canceling disturbance of different reference genes, which has been mathematically proved (27).

The thought using miRNA ratios to establish a prediction system for disease evaluation began in 2011. Mattia Boeri et al. performed a miRNA ratio signature study and found that signatures of miRNA ratios could reflect the prognosis of lung cancer patients with an AUC value >0.85 (28). However, the small sample size and a lack of demonstration of theoretical rationality largely weaken the confidence of their results. Then, Yu et al. mathematically verified miRNA-pair ratios to be independent of internal or external controls, which means miRNA ratios would remain stable no matter what inference genes were chosen (27). Thus, using miRNA-pair ratios instead of miRNA quantification could fully eliminate both experimental processes derived deviations and sample specific deviations. Here, for the first time, we constructed a full pipeline by using miRNA-pair ratio to construct prediction models for cancer diagnosis. Enrolled three independent cohorts with both miRNA profiling and RT-qPCR data, we rigorously proved the robustness and cross-platform stability of this approach.

Our results suggested that a 4-miRNA-pair model could well differentiate CC patients from healthy controls with an AUC of 0.78. Stability is the major advantage of this algorithm. No matter which detection method was adopted and which reference gene was chosen, miRNA ratios would remain stable in a certain sample. Here we found that our prediction models could stay effective in both miRNA chip verification and RT-qPCR validation. This cross-platform property makes it more easily to be clinical applied, creates an opportunity for its further application in new detection methods such as microfluidics and thermophoresis.

Although we have assessed this new method in the field of cancer diagnosis and achieved good classifier ability, there were still many other fields worth exploring such as distinguishing chemotherapy/radiotherapy-sensitive CC patients, diagnosis of other cancers or noncancerous diseases, monitoring therapeutic responses. Moreover, although we have analyzed the potential pathways and biological processes involved in these miRNAs, inner connections and biological interaction between those miRNA-pairs still need in-depth investigation.

In conclusion, we have established a cross-platform prediction model robust against sample-specific disturbance, which is not only well-performed in predicting CC but also promising in the diagnosis of other diseases.

## Data Availability Statement

The original contributions presented in the study are included in the article/**Supplementary Material**. Further inquiries can be directed to the corresponding authors.

## Ethics Statement

The studies involving human participants were reviewed and approved by Beijing Friendship Hospital Ethics Committee. The patients/participants provided their written informed consent to participate in this study.

## Author Contributions

DQ, LM, and SZha conceived and designed the study. DQ, RW, and QG performed all experiments. RW, SL, and SZhu helped to collect, reformat, and analyze the primary data. DQ and LM draft the manuscript. LM and SZha proofread and revise the manuscript. All authors contributed to the article and approved the submitted version.

## Funding

This work was fully supported by National Natural Science Foundation of China (82073390, 81702314), Beijing Science and Technology Nova Program (Z191100001119128), Beijing Municipal Science and Technology Project (Z191100006619081), Beijing Municipal Administration of Hospitals’ Youth Program (QML20180108), and The Digestive Medical Coordinated Development Center of Beijing Municipal Administration of Hospitals (XXZ02, XXZ01).

## Conflict of Interest

The authors declare that the research was conducted in the absence of any commercial or financial relationships that could be construed as a potential conflict of interest.

## Abbreviations

CC, colon cancer; RT-qPCR, real-time quantitative polymerase chain reaction; LASSO, least absolute shrinkage and selection operator; miRNAs, microRNAs; DE, Differentially expressed; SVM, Support vector machine; ROC, Receiver operator characteristic; AUC, area under the curve.

## References

[1] Sung H Ferlay J Siegel RL Laversanne M Soerjomataram I Jemal A. Global Cancer Statistics 2020: GLOBOCAN Estimates of Incidence and Mortality Worldwide for 36 Cancers in 185 Countries. CA A Cancer J Clin (2021), caac.21660. 10.3322/caac.21660 33538338

[2] Keum N Giovannucci E. Global Burden of Colorectal Cancer: Emerging Trends, Risk Factors and Prevention Strategies. Nat Rev Gastroenterol Hepatol (2019) 16:713–32. 10.1038/s41575-019-0189-8 31455888

[3] Amin MB Greene FL Edge SB Compton CC Gershenwald JE Brookland RK. The Eighth Edition AJCC Cancer Staging Manual: Continuing to Build a Bridge From a Population-Based to a More “Personalized” Approach to Cancer Staging. CA Cancer J Clin (2017) 67:93–9. 10.3322/caac.21388 28094848

[4] Pawlik TM. Colon Cancer. Surg Oncol Clin N Am (2018) 27:xiii–xiv. 10.1016/j.soc.2017.11.013 29496098

[5] Melki G Ghrewati M Mohamed H Barham S Kapoor A Ayoub F. Re-Educating Residents About non-Invasive Colorectal Cancer Screening: an Approach to Improving Colon Cancer Screening Compliance. Gastroenterol Res (2019) 12:312–4. 10.14740/gr1205 PMC6879031 31803311

[6] McDermott AM Kerin MJ Miller N. Identification and Validation of Mirnas as Endogenous Controls for RQ-PCR in Blood Specimens for Breast Cancer Studies. PLoS One (2013) 8:e83718. 10.1371/journal.pone.0083718 24391813 PMC3877087

[7] Schwarzenbach H da Silva AM Calin G Pantel K. Data Normalization Strategies for Microrna Quantification. Clin Chem (2015) 61:1333–42. 10.1373/clinchem.2015.239459 PMC4890630 26408530

[8] Yokoi A Matsuzaki J Yamamoto Y Yoneoka Y Takahashi K Shimizu H. Integrated Extracellular Microrna Profiling for Ovarian Cancer Screening. Nat Commun (2018) 9:4319. 10.1038/s41467-018-06434-4 30333487 PMC6192980

[9] Urabe F Matsuzaki J Yamamoto Y Kimura T Hara T Ichikawa M. Large-Scale Circulating Microrna Profiling for the Liquid Biopsy of Prostate Cancer. Clin Cancer Res (2019) 25:3016–25. 10.1158/1078-0432.CCR-18-2849 30808771

[10] Slattery ML Herrick JS Pellatt DF Stevens JR Mullany LE Wolff E. Microrna Profiles in Colorectal Carcinomas, Adenomas and Normal Colonic Mucosa: Variations in Mirna Expression and Disease Progression. Carcinogenesis (2016) 37:245–61. 10.1093/carcin/bgv249 PMC4766359 26740022

[11] Ritchie ME Phipson B Wu D Hu Y Law CW Shi W. Limma Powers Differential Expression Analyses for RNA-Sequencing and Microarray Studies. Nucleic Acids Res (2015) 43:e47–7. 10.1093/nar/gkv007 PMC4402510 25605792

[12] Friedman J Hastie T Tibshirani R. Regularization Paths for Generalized Linear Models Via Coordinate Descent. J Stat Softw (2010) 33:1–22. 20808728 PMC2929880

[13] Sachs MC. Plotroc: a Tool for Plotting ROC Curves. J Stat Softw (2017) 79:2. 10.18637/jss.v079.c02 30686944 PMC6347406

[14] Lewis BP Burge CB Bartel DP. Conserved Seed Pairing, Often Flanked by Adenosines, Indicates That Thousands of Human Genes are Microrna Targets. Cell (2005) 120:15–20. 10.1016/j.cell.2004.12.035 15652477

[15] Yu G Wang L-G Han Y He Q-Y. Clusterprofiler: an R Package for Comparing Biological Themes Among Gene Clusters. OMICS (2012) 16:284–7. 10.1089/omi.2011.0118 PMC3339379 22455463

[16] Kanehisa M Goto S. KEGG: Kyoto Encyclopedia of Genes and Genomes. Nucleic Acids Res (2000) 28:27–30. 10.1093/nar/28.1.27 10592173 PMC102409

[17] Jung G Hernández-Illán E Moreira L Balaguer F Goel A. Epigenetics of Colorectal Cancer: Biomarker and Therapeutic Potential. Nat Rev Gastroenterol Hepatol (2020) 17:111–30. 10.1038/s41575-019-0230-y PMC7228650 31900466

[18] Cojocneanu R Braicu C Raduly L Jurj A Zanoaga O Magdo L. Plasma and Tissue Specific Mirna Expression Pattern and Functional Analysis Associated to Colorectal Cancer Patients. Cancers (2020) 12:843. 10.3390/cancers12040843 PMC7226631 32244548

[19] Vychytilova-Faltejskova P Radova L Sachlova M Kosarova Z Slaba K Fabian P. Serum-Based Microrna Signatures in Early Diagnosis and Prognosis Prediction of Colon Cancer. Carcinogenesis (2016) 37:941–50. 10.1093/carcin/bgw078 27485599

[20] Giráldez MD Lozano JJ Ramírez G Hijona E Bujanda L Castells A. Circulating Micrornas as Biomarkers of Colorectal Cancer: Results From a Genome-Wide Profiling and Validation Study. Clin Gastroenterol Hepatol (2013) 11:681–8.e3. 10.1016/j.cgh.2012.12.009 23267864

[21] Donati S Ciuffi S Brandi ML. Human Circulating Mirnas Real-Time Qrt-PCR-Based Analysis: an Overview of Endogenous Reference Genes Used for Data Normalization. Int J Mol Sci (2019) 20:4353. 10.3390/ijms20184353 PMC6769746 31491899

[22] Xiang M Zeng Y Yang R Xu H Chen Z Zhong J. U6 is Not a Suitable Endogenous Control for the Quantification of Circulating Micrornas. Biochem Biophys Res Commun (2014) 454:210–4. 10.1016/j.bbrc.2014.10.064 25450382

[23] Ge W Yu D-C Li Q-G Chen X Zhang C-Y Ding Y-T. Expression of Serum Mir-16, Let-7f, and Mir-21 in Patients With Hepatocellular Carcinoma and Their Clinical Significances. Clin Lab (2014) 60:427–34. 10.7754/clin.lab.2013.130133 24697119

[24] Chugh P Dittmer DP. Potential Pitfalls in Microrna Profiling. Wiley Interdiscip Rev RNA (2012) 3:601–16. 10.1002/wrna.1120 PMC3597218 22566380

[25] Benz F Roderburg C Vargas Cardenas D Vucur M Gautheron J Koch A. U6 is Unsuitable for Normalization of Serum Mirna Levels in Patients With Sepsis or Liver Fibrosis. Exp Mol Med (2013) 45:e42. 10.1038/emm.2013.81 24052167 PMC3789266

[26] Occhipinti G Giulietti M Principato G Piva F. The Choice of Endogenous Controls in Exosomal Microrna Assessments From Biofluids. Tumour Biol (2016) 37:11657–65. 10.1007/s13277-016-5164-1 27438704

[27] Deng Y Zhu Y Wang H Khadka VS Hu L Ai J. Ratio-Based Method to Identify True Biomarkers by Normalizing Circulating Ncrna Sequencing and Quantitative PCR Data. Anal Chem (2019) 91:6746–53. 10.1021/acs.analchem.9b00821 PMC6884007 31002238

[28] Boeri M Verri C Conte D Roz L Modena P Facchinetti F. Microrna Signatures in Tissues and Plasma Predict Development and Prognosis of Computed Tomography Detected Lung Cancer. Proc Natl Acad Sci U S A (2011) 108:3713–8. 10.1073/pnas.1100048108 PMC3048155 21300873

