# Supplementary material for: Predict Colon Cancer by Pairing Plasma miRNAs: Establishment of a Normalizer-Free, Cross-Platform Model
Source: Front Oncol. 2021 Apr 22;11:561763. doi: 10.3389/fonc.2021.561763 (PMC8101326; doi:10.3389/fonc.2021.561763)
Supplement: Supplementary file 1 [file Table_1.docx]

**Supplement File 1. Clinical information of 104 participants**

| *Clinical information* | | *Normal control group*  *N=48* | *Early CC group*  *N=16* | *Advanced CC group*  *N=40* |
| --- | --- | --- | --- | --- |
| *Gender* | Male | 36(75%) | 13(82%) | 31(78%) |
|  | Female | 12(25%) | 3(18%) | 9(22%) |
| *Age* | < 60 | 28(58%) | 7(44%) | 17(43%) |
|  | > 60 | 20(42%) | 9(56%) | 23(57%) |
| *Location* | Left colon | / | 7(44%) | 16(40%) |
|  | Right colon | / | 5(31%) | 8(20%) |
|  | Rectum | / | 2(13%) | 14(35%) |
| *Size* | > 2cm | / | 5(31%) | 28(70%) |
|  | < 2cm | / | 11(69%) | 8(20%) |
