# Supplementary material for: Predict Colon Cancer by Pairing Plasma miRNAs: Establishment of a Normalizer-Free, Cross-Platform Model
Source: Front Oncol. 2021 Apr 22;11:561763. doi: 10.3389/fonc.2021.561763 (PMC8101326; doi:10.3389/fonc.2021.561763)
Supplement: Supplementary file 2 [file Table_2.docx]

**Supplement File 2. Details of the primers used for RT-qPCR validation**

RT-primers and forward primers were shown as follows. Universal reverse primer was used in q-PCR of every miRNA in this study.

hsa-miR-1246-RT:

GTCGTATCCAGTGCAGGGTCCGAGGTATTCGCACTGGATACGACCCTGCT

hsa-miR-1246-Forward:

GCTGGAATGGATTTTTGG

hsa-miR-451a-RT:

GTCGTATCCAGTGCAGGGTCCGAGGTATTCGCACTGGATACGACAACTCA

hsa-miR-451a- Forward:

GCCGAAACCGTTACCATTAC

hsa-miR-4514-RT:

GTCGTATCCAGTGCAGGGTCCGAGGTATTCGCACTGGATACGACTTCCCC

hsa-miR-4514-Forward:

CGGCACAGGCAGGATT

hsa-miR-654-5p-RT:

GTCGTATCCAGTGCAGGGTCCGAGGTATTCGCACTGGATACGACGCACAT

hsa-miR-654-5p-Forward:

TGGTGGGCCGCAGAAC

hsa-miR-575-RT:

GTCGTATCCAGTGCAGGGTCCGAGGTATTCGCACTGGATACGACGCTCCT

hsa-miR-575-Forward:

GCGGAGCCAGTTGGAC

hsa-miR-4299-RT:

GTCGTATCCAGTGCAGGGTCCGAGGTATTCGCACTGGATACGACGCCTCT

hsa-miR-4299-Forward:

GCTGGGCTGGTGACATG

Universal Reverse Primer:

AGTGCAGGGTCCGAGGT
